# HER2-low breast cancer in routine practice: a nationwide study of diagnostic variability across pathology laboratories

**DOI:** 10.1007/s00428-025-04266-4

**Published:** 2025-09-16

**Authors:** Zeynep E. Kain, Ximena Baez-Navarro, Nils A. ’t Hart, Carolien H. M. van Deurzen

**Affiliations:** 1 Department of Pathology, Erasmus MC, Rotterdam, The Netherlands; 2 Department of Pathology, Isala Klinieken, Zwolle, The Netherlands

**Keywords:** Breast cancer, HER2-low, Immunohistochemistry, Interlaboratory variability, Antibody

## Abstract

Patients with HER2-low breast cancer (BC) may be eligible for trastuzumab-deruxtecan (T-DXd) treatment. However, studies have shown that different HER2 antibodies vary in their sensitivity for low HER2 expression, potentially impacting HER2-low BC diagnosis and patient selection for T-DXd. We investigated the frequency of HER2-low BC in relation to the HER2-antibody used across Dutch pathology laboratories. Patients with primary BC without neoadjuvant treatment, diagnosed between 2013 and 2024, were included. HER2-low frequencies from 34 laboratories were obtained from the Dutch Nationwide Pathology Databank (Palga). Additional information (e.g., type of HER2 antibody, staining protocol) was obtained through a questionnaire. A total of 88,713 patients were included, representing 103,505 tumors, of which 94,934 had a conclusive HER2 status. Among non-amplified cases, HER2-low frequencies varied widely across laboratories (33.4%–94.5%), with a gradual increase since 2022. The most commonly used antibody clones were 4B5 (*n* = 21), DG44 (*n* = 7), A0485 (*n* = 4), and SP3 (*n* = 2). HER2-low proportions were highest with A0485 (71.5%), followed by DG44 (66.7%), SP3 (60.1%), and 4B5 (59.1% with Ultraview, 57.0% with Optiview). Substantial inter-laboratory variation was observed even within the same antibody group (4B5/Ultraview: 40.5%–80.4%; 4B5/Optiview: 37.3%–68.4%; DG44: 40.6%–95.4%; A0485: 62.3%–94.7%; SP3: 31.6%–78.6%). Our data showed a notable variation in HER2-low BC frequency across Dutch pathology laboratories, even among those using the same antibody and detection system. These differences may influence patient eligibility for T-DXd.

## Introduction

Breast cancer (BC) is the most common malignancy among women worldwide [1]. For treatment purposes, BCs are subtyped based on the expression of estrogen receptor (ER), progesterone receptor (PgR) and the Human epidermal growth factor receptor 2 (HER2). Until recently, HER2 was classified dichotomous as either negative or positive for treatment purposes, based on protein overexpression and/or amplification [2].

The humanized monoclonal antibody Trastuzumab (Herceptin) showed significant therapeutic effects in HER2-positive BC [3, 4]. More recently, antibody–drug conjugates (ADCs) have expanded treatment options for BC patients with low levels of HER2 expression (HER2-low). The DESTINY-Breast04 clinical trial, involving patients with HER2-low metastatic BC (defined as HER2 score 1 + on immunohistochemical (IHC) analysis or IHC score 2 + without gene amplification on in situ hybridization (ISH)), demonstrated notable improvements in progression-free survival (PFS) and overall survival (OS) in patients treated with T-DXd compared to conventional chemotherapy agents [5, 6]. Later, in the DESTINY-Breast06 trial, the significant effect of T-DXd was also demonstrated in patients with HER2-ultralow tumors [7, 8]. In January 2025, the FDA approved T-DXd for the treatment of patients with unresectable or metastatic hormone receptor (HR)-positive, HER2-low and HER2-ultralow BC [9, 10]. Shortly after, the European Medicines Agency (EMA) also approved the use of T-DXd in the same patient group [11]. Given the application of this new drug, evaluation of low levels of HER2 expression has become increasingly critical for pathologists.

In the DESTINY-Breast04 trial, the VENTANA HER2/neu (4B5) IUO (investigational use only) assay system with Ultraview detection kit was used to evaluate HER2 IHC scores. Although the optimal lower threshold for HER2 protein detection is unknown, this resulted in the FDA recommendation to use the 4B5 antibody to determine HER2-low BC. In contrast, the EMA does not mandate to use a specific antibody for HER2-low detection [12].

Several studies compared different HER2 antibodies with inconsistent results [13–16]. Ruschoff et al. compared HercepTest monoclonal antibody (mAb) pharmDx (clone DG44) with PATHWAY 4B5, while Layfield et al. and Karakas et al. compared HercepTest polyclonal antibody (pAb) (clone A0485) with PATHWAY 4B5 and they demonstrated that HercepTest is more sensitive to detect HER2-low cases [13–15]. Inversely, Scott et al. demonstrated that the PATHWAY 4B5 assay identified a higher proportion of HER2-low cases compared to HercepTest, suggesting superior sensitivity in detecting HER2-low expression [16]. Hempenius et al. demonstrated that clone DG44 and 4B5 Optiview had a lower limit of detection compared to SP3 and 4B5 Ultraview, indicating their high sensitivity [17].

The use of different HER2 antibodies across pathology laboratories in daily practice could impact the HER2-low rate among non-amplified cases, and consequently, the selection of patients for treatment with T-DXd. In this study, we aimed to assess the interlaboratory variability in HER2-low assessment across Dutch pathology laboratories.

## Material and methods

### Data acquisition

A retrospective analysis was performed, including all primary invasive BC resection specimens reported via the Dutch Nationwide Pathology Databank (Palga) between January 1, 2013, and December 31, 2024 [18]. Patients older than 18 years who did not receive neoadjuvant treatment for BC were included. Multiple tumors and/or second primary tumors after the first diagnosis counted as separate cases. We collected clinical characteristics, such as sex, age at diagnosis, type of surgery and histopathologic features of the resection specimen including histologic subtype, tumor diameter, grade, angio-invasion and ER/PgR status.

In the synoptic reporting module of Palga, it is mandatory to provide detailed information regarding the HER2 testing method and the subsequent results. This includes the tissue type used for testing (needle biopsy or resection specimen), along with the immunohistochemistry (IHC) result (scored as 0, 1 +, 2 +, or 3 +) based on the American Society of Clinical Oncology/College of American Pathologists (ASCO/CAP) guidelines [8]. Additionally, the type of reflex test was indicated, such as chromogenic in situ hybridization, silver in situ hybridization, fluorescence in situ hybridization, or PCR. HER2 status was determined according to the established international guidelines available at the time of diagnosis [6, 19]. Combined IHC data from the biopsy and the resection specimen were used to determine the HER2 category. If these variables differed, data from the resection specimen were used. HER2 status was categorized as HER2-0, HER2-low (IHC 1 + or 2 + with a negative reflex test) and HER2-positive following the updated ASCO/CAP guideline on HER2-testing in BC [8].

In addition, an online survey was distributed to all pathology laboratories in the Netherlands regarding the HER2 IHC protocol including the brand and clone of the primary antibody, staining platform, detection kit currently in use and any changes made during the study period.

### Statistical analysis

Descriptive statistics were performed. We calculated absolute and relative values for categorical variables. Median and range as per 25th and 75th percentiles were calculated for non-normally distributed continuous variables. Statistical analyses were done in SPSS (IBM Corp. Released 2021. IBM SPSS Statistics for Windows, Version 28.0, IBM Corp).

To minimize selection bias resulting from the exclusion of patients who received neoadjuvant therapy, the analysis was limited to cases with HER2 non-amplified tumors.

## Results

Out of the 38 laboratories, 37 completed the survey and granted permission to be de-blinded in the national database. Three laboratories were excluded: two for not using Palga synoptic reporting, and one for not using HER2 IHC at all, instead applying FISH on each BC specimen.

Thirty-four pathology laboratories were included in the study. The total number of BC patients was 88,713, which resulted in 103,505 invasive BCs. The median age was 65 years (range 19–101 years), 99.2% (*n* = 87,958) were female and 0.8% (*n* = 728) were male. Most patients (64.5%, n = 66,778) underwent breast-conserving surgery, while 35.1% (n = 36,387) underwent mastectomy. The median tumor diameter was 1.4 cm (range 0.01–23.0 cm). Most tumors were classified as no-special type (NST) (77%, *n* = 80,150), Bloom-Richardson grade 2 (49.6%, *n* = 51,298), showed no angio-invasion 81.3% (*n* = 84,177), and had an ER-positive status (88%, *n* = 91,123).

### General HER2 findings

Both HER2 IHC score and final HER2 status (amplified versus non-amplified) were available in 91% (*n* = 94,934) of the cases. HER2 IHC scores were: 35.6% (*n* = 33,012) score 0, 41% (*n* = 38,074) score 1 +, 17.2% (*n* = 15,975) score 2 + and 6.1% (*n* = 5692) score 3 +. After reflex testing, this resulted in the following categories: 36% (*n* = 33,012) of the cases were HER2-0, 56.7% (*n* = 52,664) were HER2-low and 7.6% (*n* = 7077) were HER2-positive.

### Variation in general HER2 findings

**Fig. 1:**
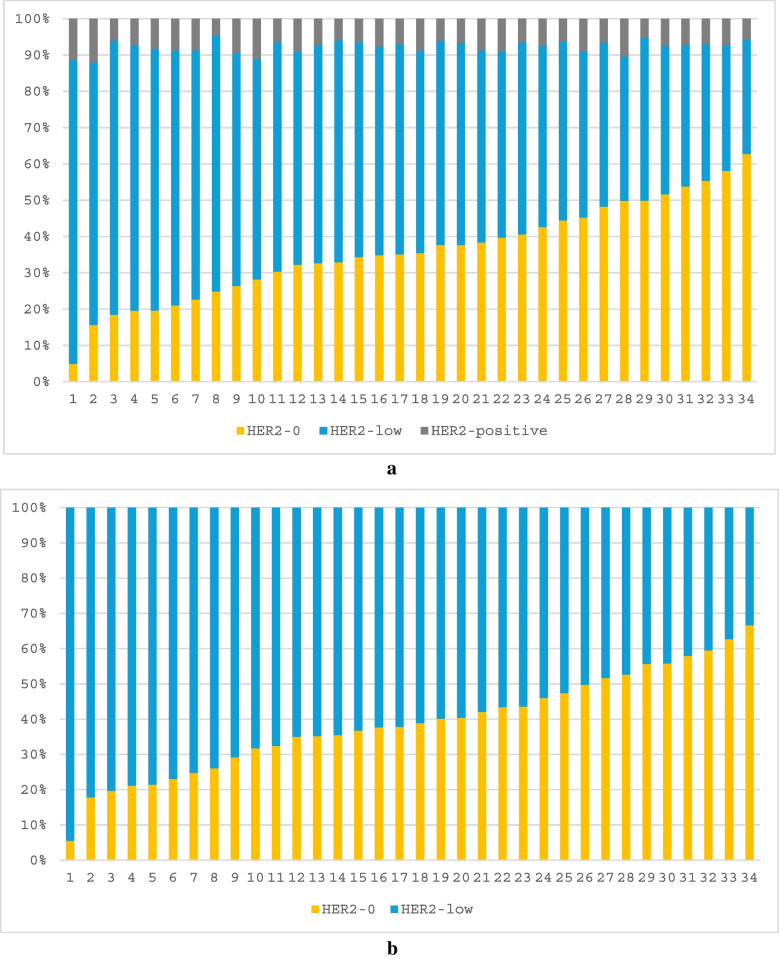
**a** Distribution of HER2-levels (HER2-0 versus HER2-low versus HER2-amplified) of BC cases per pathology laboratory. **b** Distribution of HER2-levels (HER2-0 versus HER2-low) among non-amplified BC cases per pathology laboratory

The total number of cases per laboratory ranged from 635 to 7075 (median 2294). The distribution of HER2-low also varied across laboratories with percentages ranging from 31 to 83% (Fig. 1a). Among non-amplified cases, the HER2-low frequency ranged from 33.4% to 94.5% (Fig. 1b).

**Fig. 2:**
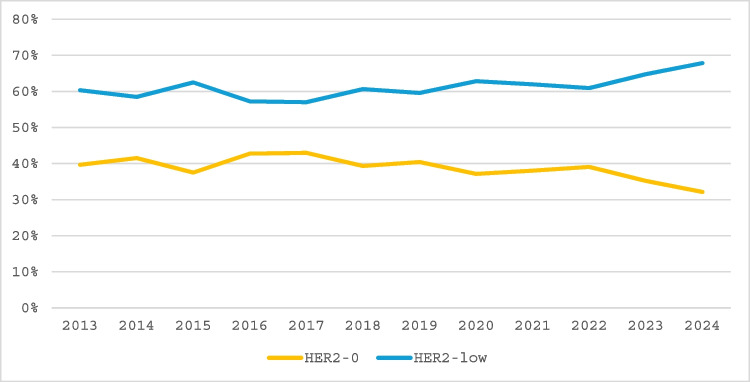
Distribution of HER2-0 and HER2-low rates per year, limited to non-amplified BC cases

Until 2022, the HER2-low rates were relatively stable, fluctuating around 60%. However, since 2022, there is a gradual increase in the frequency of HER2-low cases up to 67.8% in 2024 (Fig. 2).

### HER2 antibodies used across laboratories

Among the 34 laboratories, four antibody clones were primarily used: 4B5 (Ventana, PATHWAY® or Optiview), HercepTest™ mAb pharmDx (clone DG44, Dako Omnis), (concentrated) polyclonal anti-human c-erbB2 antibody (clone A0485, Dako Agilent), and HER2/neu monoclonal anti-erbB2 antibody (clone SP3, Cell Marque).

Currently, the most used clone is 4B5 (*n* = 21 laboratories; 60%), followed by DG44 (*n* = 7 laboratories; 20%), A0485 (*n* = 4 laboratories; 15%), and SP3 (*n* = 2 laboratories; 5%). Of the laboratories using 4B5, 17 utilize the Ultraview detection kit, while 4 use the Optiview detection kit. Over the years, seven laboratories have switched clones. Three laboratories switched from A0485 to DG44, two laboratories switched from SP3 to DG44, one laboratory moved from SP3 to 4B5-Optiview, one laboratory changed from clone CB11 (The BOND Oracle HER2 IHC assay, Leica Biosystems) to DG44.

### Association between HER2-antibody and frequency of HER2-low

**Fig. 3:**
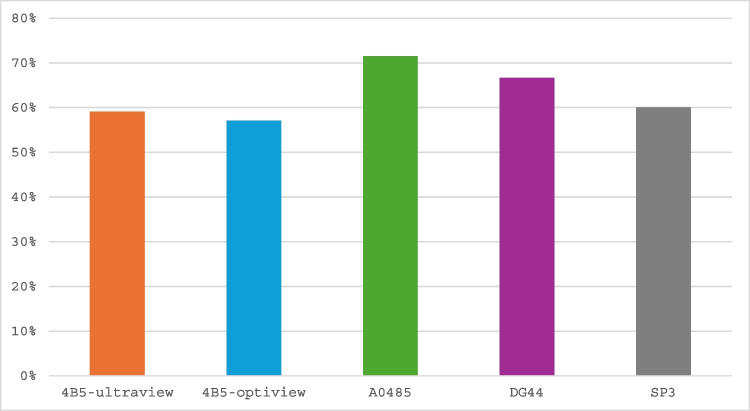
Average frequency of HER2-low BC among non-amplified cases, per antibody

There was a correlation between the HER2-low rate and the used HER2 antibody clone. Among non-amplified cases, the highest average HER2-low rate was seen in laboratories using the A0485 clone (71.5%), followed by laboratories using DG44 (66.7%). The other antibodies had a comparable HER2-low rate (60.1% for SP3, 59.1% for 4B5/Ultraview, 57% for 4B5/Optiview, 58.2% for CB11 (Fig. 3; CB11 is excluded since this was only used in the past in one laboratory). One laboratory stained 64 cases with a (not-concentrated) Polyclonal antibody (HercepTest™ K5204) before 2018. This number of cases was insufficient to determine a meaningful HER2-low percentage and was therefore excluded from this figure.

### Variation between laboratories using the same antibody

**Fig. 4:**
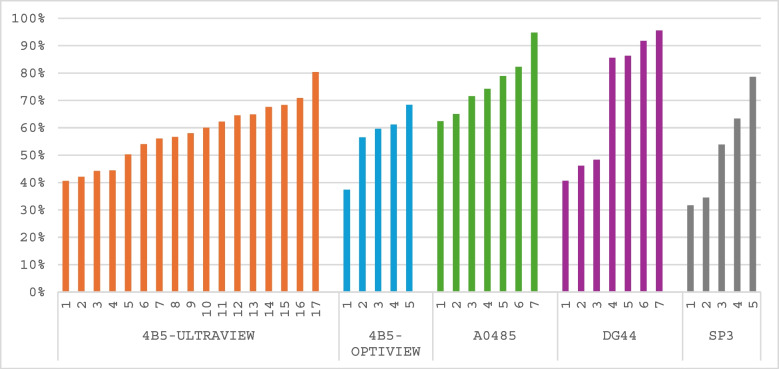
HER2-low rates among non-amplified cases across the laboratories, grouped according to the used HER2 antibody

To evaluate inter-laboratory variability of the laboratories using the same antibody, we analyzed the HER2-low rate among laboratories using the same antibody. Figure 4 presents the HER2-low rates among non-amplified cases per antibody per laboratory.

All laboratories using clone 4B5 have the same staining platform (the Ventana Benchmark Ultra) and follow the manufacturer’s manuals and protocols. They used either the Ultraview or the Optiview detection kit (*n* = 17 and *n* = 5, respectively). Among the laboratories using 4B5 Optiview, the HER2-low rate ranged between 37.3% and 68.4%. The HER2-low range was 40.5% to 80.4% in laboratories using 4B5 Ultraview. In laboratories, using A0485, DG44 and SP3, the range was also very large (62.4% to 94.7% for A04B5, 40.6% to 95.5% for DG44 and 31.7% to 78.6% for SP3), as illustrated in Fig. 4.

It is important to note that different staining protocols were employed by each laboratory for antibodies other than 4B5, which may influence the comparability of the results.

### Laboratories with an antibody switch and the effect on HER2-low frequency

One laboratory that utilizes the Ventana 4B5 antibody switched from the Optiview to the Ultraview detection kit in March 2024. The rate of HER2-low was not substantially different before and after this change (58.4% versus 56%).

One lab switched from clone A0485 to DG44 in 2021. The rates of HER2-low changed from 71.5% to 85.5%. One lab switched from SP3 to the 4B5 clone with the OptiView detection kit in 2021. The rates of HER2-low increased from 34.5% to 68.3%.

## Discussion

The introduction of T-DXd for patients with HER2-low BC increased the relevance of quantifying low levels of HER2. Previous studies demonstrated that different HER2-antibodies have varying detection limits of HER2 protein detection, potentially affecting the diagnosis of HER2-low BC, and consequently, the selection of patients for treatment with T-DXd [17, 20]. In this nationwide study, including 34 laboratories, we investigated the frequency of HER2-low BC in relation to the HER2-antibody used across pathology laboratories.

Our results showed that the HER2-low rate gradually increased since 2022. In the Netherlands, T-DXd is not included in standard care yet, but ongoing clinical trials and the growing attention to HER2-low in the literature and at conferences likely resulted in increased awareness among pathologists. This may have resulted in pathologists being more likely to classify a BC as HER2-low. The HER2-amplification rate in this study is relatively low (<10%), which is likely due to the exclusion of patients who received neoadjuvant therapy, among whom HER2-positive cases are more prevalent.

We demonstrated that there is considerable interlaboratory variation in HER2 assessment in the Netherlands. The overall frequency of HER2-low ranged from 31.4 to 83.7% among non-amplified cases. These results are slightly compatible with the result of a nationwide Danish study of Nielsen et al. [21] and a recent global study [22]. This substantial interlaboratory variability may have multiple causes, including the use of different antibody assays/staining protocols and the use of inconsistent scoring approaches by different pathologists. In our study, we also compared the rates of HER2-0 vs. HER2-low across the laboratories using the same HER2 antibody and staining platform, which also showed significant differences between the laboratories. The HER2-low rate varied from 40.5% to 80.4% across the laboratories utilizing PATHWAY 4B5, suggesting that a significant proportion of scoring variability is attributed to differences in interpretation among pathologists.

Several studies investigated the interobserver variation between HER2 antibodies regarding HER2-low BC scoring, with conflicting results. Ruschoff et al. found slightly higher interobserver agreement with PATHWAY 4B5 compared to the HercepTest mAb (clone DG44) [14]. On the other hand, Layfield et al. reported a higher interobserver agreement with HercepTest pAb (clone A0485) compared to 4B5 (κ = 0.74 vs. 0.57) [13]. Karakas et al. also demonstrated higher agreement rates and Kappa values with HercepTest pAb (A0485) (κ = 0.76 vs. 0.65) compared to PATHWAY 4B5, especially in distinguishing 1 + from 2 + scores [15].

In addition to interobserver variability, the choice of antibody assay could also influence HER2 status determination. For example, one laboratory in our cohort switched from the A0485 polyclonal antibody to the DG44 monoclonal antibody in 2021. This change was associated with an increase in the rate of HER2-low cases. However, we cannot exclude the possibility that this was the result of other variables, including the growing clinical awareness of the HER2-low category during that period.

Several studies evaluated the sensitivity of different HER2 antibody clones, also with conflicting results. Ruschoff et al. showed that HercepTest mAb (clone DG44) was more sensitive to detect HER2-low cases in comparison with 4B5 [14]. Supporting this, the NordiQC 2024 external quality assessment round demonstrated that HercepTest mAb (clone DG44) showed the highest performance, with a 100% pass rate and 61% optimal results, outperforming PATHWAY 4B5 [23]. In contrast, in a cohort of 500 BC patients, Scott et al. reported that the PATHWAY 4B5 demonstrated slightly higher detection rates of HER2-low cases compared to the HercepTest [16]. The use of new objective assays to quantify the amount of HER2 protein could improve the standardization of HER2-low testing across laboratories [24, 25].

The main strength of our study is its use of real-world, population-based data collected from 34 laboratories. However, this study also has limitations, inherent to all large-scale retrospective studies: there was no central pathology review. Since HER2 testing was optimized to identify HER2-amplified cases during the study period, with a lack of clinical relevance of differentiation between HER2-0 and HER2-low, it is likely that pathologist had less attention for the precise scoring (HER2-0 versus HER2 1 +) among the HER2 non-amplified cases.

In conclusion, we compared the incidence of HER2-low BC among pathology laboratories in the Netherlands. The overall HER2-low rate was 56.8% among non-amplified cases, with a range from 33.4% to 94.5%. Notably, even among laboratories using the same antibody clone and standardized protocols, HER2-low rates still varied significantly. These findings highlight the need for further standardization in HER2 testing and scoring, to optimize the selection of patients for treatment with T-DXd.

## Data Availability

The datasets used for this study are available from the corresponding author upon reasonable request.
